# *FPGS* relapse-specific mutations in relapsed childhood acute lymphoblastic leukemia

**DOI:** 10.1038/s41598-020-69059-y

**Published:** 2020-07-21

**Authors:** Sung-Liang Yu, Hui Zhang, Bing-Ching Ho, Chih-Hsiang Yu, Chia-Ching Chang, Yin-Chen Hsu, Yu-Ling Ni, Kai-Hsin Lin, Shiann-Tarng Jou, Meng-Yao Lu, Shu-Huey Chen, Kang-Hsi Wu, Shih-Chung Wang, Hsiu-Hao Chang, Ching-Hon Pui, Jun J. Yang, Jinghui Zhang, Dong-Tsamn Lin, Shu-Wha Lin, Xiaotu Ma, Yung-Li Yang

**Affiliations:** 1 Centers of Genomic and Precision Medicine, National Taiwan University, Taipei, Taiwan; 2 Department of Clinical Laboratory Sciences and Medical Biotechnology, College of Medicine, National Taiwan University, Taipei, Taiwan; 3 Department of Laboratory Medicine, National Taiwan University Hospital and National Taiwan University College of Medicine, No. 7, Chung Shan S. Rd, Zhongshan S. Rd, Zhongzheng Dist, Taipei, 100 Taiwan, ROC; 4 Institute of Medical Device and Imaging, College of Medicine, National Taiwan University, Taipei, Taiwan; 5 Graduate Institute of Pathology, College of Medicine, National Taiwan University, Taipei, Taiwan; 6 Graduate Institute of Clinical Medicine, National Taiwan University College of Medicine, Taipei, Taiwan; 7 Department of Hematology & Oncology, Guangzhou Women and Children’s Medical Center, Guangzhou, Guangdong China; 8 Department of Pediatrics, National Taiwan University Hospital and National Taiwan University College of Medicine, Taipei, Taiwan; 9 Department of Pediatrics, Taipei Medical University–Shuang Ho Hospital, Taipei, Taiwan; 10 Division of Pediatric Hematology &Oncology, China Medical University Children’s Hospital, Taichung, Taiwan; 11 Department of Pediatrics, Changhua Christian Hospital, Changhua, Taiwan; 12 Department of Oncology, St. Jude Children’s Research Hospital, Memphis, TN USA; 13 Department of Pharmaceutical Sciences, St. Jude Children’s Research Hospital, Memphis, TN USA; 14 Department of Computational Biology, St. Jude Children’s Research Hospital, Memphis, TN USA

**Keywords:** Cancer genomics, Haematological cancer, Paediatric cancer

## Abstract

Although the cure rate for childhood acute lymphoblastic leukemia (ALL) has exceeded 80% with contemporary therapy, relapsed ALL remains a leading cause of cancer-related death in children. Relapse-specific mutations can be identified by comprehensive genome sequencing and might have clinical significance. Applying whole-exome sequencing to eight triplicate samples, we identified in one patient relapse-specific mutations in the *folylpolyglutamate synthetase* (*FPGS*) gene, whose product catalyzes the addition of multiple glutamate residues (polyglutamation) to methotrexate upon their entry into the cells. To determine the prevalence of mutations of the *FPGS* mutations, and those of two important genes in the thiopurine pathway, *NT5C2* and *PRPS1*, we studied 299 diagnostic and 73 relapsed samples in 372 patients. Three more *FPGS* mutants were identified in two patients, *NT5C2* mutations in six patients, and *PRPS1* mutants in two patients. One patient had both *NT5C2* and *PRPS1* mutants. None of these alterations were detected at diagnosis with a sequencing depth of 1000X, suggesting that treatment pressure led to increased prevalence of mutations during therapy. Functional characterization of the *FPGS* mutants showed that they directly resulted in decreased enzymatic activity, leading to significant reduction in methotrexate polyglutamation, and therefore likely contributed to drug resistance and relapse in these cases. Thus, besides genomic alterations in thiopurine metabolizing enzymes, the relapse-specific mutations of *FPGS* represent another critical mechanism of acquired antimetabolite drug resistance in relapsed childhood ALL.

## Introduction

Acute lymphoblastic leukemia (ALL), the most common childhood cancer, accounts for approximately 25% of pediatric maligancies^1^. Despite overall 5-year event-free survival rates have increased to 80% or more in the developed countries with the use of improved risk-directed treatment and supportive care, as many as 10–20% of patients still suffered from relapse^2–10^. Most of the relapsed patients, especially those with early recurrence, died even with aggressive salvage strategies including allogeneic hematopoietic cell transplantation^11,12^. Understanding the mechanism of relapse would allow revisions of current front-line clinical trials to circumvent this adverse event. Current comprehensive genome sequencing studies suggested that leukemia cells of most relapsed patients, especially those with late recurrence, acquired new secondary genetic alterations, usually within a minor clone arising after diagnosis of ALL^13–15^.

Thiopurines (6-mercaptopurine (6-MP) and 6-thioguanine (6-TG)) are key components of maintenance therapy for ALL in both adults and children^2^. Thiopurines are enzymatically converted to with cytotoxic thioguanine nucleotides inside the cell, and acquired mutations in the purine salvage pathway genes have been associated with thiopurine resistance in relapsed ALL (*NT5C2*, *PRPS1*)^16–18^. MTX is another essential chemotherapeutic for the curative treatment of ALL^2^. The cytotoxic activity of MTX is related to the activity of FPGS (folylpolyglutamate synthetase), which activates MTX by polyglutamation^19^. The ability of leukemic cells to retain MTX polyglutamates has been associated with MTX response in vivo in ALL patients^20^. Disruption of FPGS function can confer MTX resistance and compromise activity of other polyglutamylation-dependent antifolates in leukemia cell lines as well as in primary ALL blasts^20^. Moreover, concomitant use of MTX increases the conversion of 6MP to thioguanine nucleotides^21^, Therefore, decreased MTX polyglutamylation can adversely affect antileukemic efficacy of MTXbut also 6MP activity.

In a study of whole-exome sequencing of eight matched diagnosis-remission-relapse triplicate ALL samples, we identified a case that acquired a novel point mutation of *FPGS* at relapse. We then sequenced another 73 relapsed samples and identified three additional genetic alterations of *FPGS*. Further analysis showed that these *FPGS* mutants had lost their polyglutamylation function by comparison with that of cells expressing the wide type gene. Hence, in addition to mutant enzymes in the purine pathway, acquired relapse-specific mutations in genes participating in MTX metabolism pathway also provide a biochemical mechanism of antimetabolite drug resistance in relapsed childhood ALL.

## Materials and methods

### Patients and protocols

Bone marrow samples taken at the time of diagnosis, relapse and clinical remission were available for eight patients with ALL (five boys and three girls) treated at National Taiwan University Hospital from January 2002 through December 2012. Three hundred seventy-two patients with ALL enrolled in the TPOG ALL 93, ALL 97 VHR, ALL 2002, or ALL 2013 protocols between January 1997 and December 2016 served as the validation cohort. Detailed results of the TPOG ALL 93, ALL 97 VHR and ALL 2002 protocols had been published elsewhere^22,23^. The TPOG ALL 2013 was modified from ALL 2002 by the incorporation of minimal residual disease (MRD) measurements to guide the treatment strategy. Depending on the risk stratification of the patients, high-dose methotrexate was administered at 1, 2.5 or 5 gm/m^2^ over 24 h together with daily mercaptopurine. In the maintenance phase, low-dose methotrexate was administered intramuscularly at 40 mg/m^2^ together with mercaptopurine. Genomic DNA was extracted from leukemic cells of bone marrow or peripheral blood origin by using standard phenol and chloroform-based methods. This study was approved by the institutional review board of National Taiwan University Hospital. All of the participants and/or their guardians provided written informed consent in accordance with the Declaration of Helsinki.

### Whole-exome sequencing and analysis

Whole-exome capture libraries were prepared using the SureSelect Human All Exon 50 Mb or 38 Mb kit (Agilent) with the depth of 120X. We performed paired-end (2 × 100 bp) sequencing using the Illumina HiSeq 2000 instrument, and imaging analysis and base calls using the Illumina Real Time Analysis (RTA) Pipeline software, version 1.9. After removing reads whose sequences matched the sequencing adapters as well as low-quality reads (in which more than 50% of bases had a Phred quality score < 5), we aligned the remaining reads the reference human genome (hg19, https://genome.ucsc.edu/) by Burrows–Wheeler analysis with default parameters. We then independently identified sites that differed from the reference finding in each sample. SNVs/indels were detected as previously described^24^. The reference human genome assembly GRCh37-lite was used to map all samples. For each case, somatic variants were detected by paired analysis of diagnosis-germline and relapse-germline samples. Specifically, SNV and indels were analyzed by Bambino followed by post-process filtering^25^, copy number variation by CONSERTING^26^ and structural variation by CREST^27^. Our analysis of non-coding SNVs do not include tier 4 variants (i.e. those that are present in genomic regions that contain highly repetitive elements such as Alu or Line elements).

### Copy number alteration analysis

DNA was analyzed by the SALSA multiplex ligation-dependent probe amplification (MLPA) kit (P335-A4) (MRC-Holland, Amsterdam, the Netherlands) according to suggestions of the manufacturer. This kit includes probes for *IKZF1* (7p12.2, exons 1 to 8), *CDKN2A* (9p21.3, exons 2 and 4), *CDKN2B* (9p21.3, exon 2), *PAX5* (9p13.2, exons 1, 2, 5, 6, 8, and 10), *EBF1* (5q33.3, exons 1, 10, 14, and 16), *ETV6* (12p13.2, exons 1, 2, 3, 5, and 8), *BTG1*(12.q22, exon 1 and 2), *RB1* (13q14.2, exons 6, 14, 19, 24, and 29), SHOX (Xp22.33), *CSF2RA* (Xp22.33, exon 16), *IL3RA* (Xp22.33, exon 1), and *CRLF2* (Xp22.33, exon 4). The PCR fragments were separated by capillary electrophoresis on a Life Technologies 3,500 Genetic Analyzer (Thermo Fisher Scientific, Waltham, MA). MLPA data were analyzed with an ABI 3,730 capillary sequencer (Applied Biosystems, Foster City, CA). The relative copy number was determined after the normalization of peaks against controls. Values between 0.75 and 1.3 were considered within the normal range. Values below 0.75 or above 1.3 indicated deletion or gain, respectively. Values below 0.25 indicated biallelic deletion^28,29^.

### Deep sequencing

*FPGS*, *NT5C2* and *PRPS1* genes were first applied to custom GeneRead DNAseq Targeted Panel v2 design (Qiagen) according to manufacturer's instructions. The custom GeneRead DNAseq Targeted Panel included 73 amplicons covering the exon regions of *FPGS*, *NT5C2* and *PRPS1* genes and divided into three separate pools. Ten ng genomic DNA was used for multiplex PCR, and the resulting PCR products underwent library preparation and indexing using KAPA LTP Library Preparation kit (Kapa Biosystems) according to the manufacturer’s protocol. For target sequencing, libraries were pooled with equal molarity and sequenced on the Illumina NextSeq500 instrument with the 300 cycle Reagent kit (Illumina). The depth of deep sequencing was more than 1000X. Details of the primers’ sequences are listed in the Supplementary Table 1.

### Variant calling of target sequencing

To identify variants, we first converted and demultiplexed BCL basecall files generated by Illumina sequencing to fastq files using the bcl2 fastq tool according to the user guide (v2.15, Illumina). The fastq files were mapped to the GRCh37 build of the human genome reference using BWA-MEM (v0.7.15-r1140). Mapped reads in SAM format were then compressed to the BAM format by Samtools (v1.3.1). The base quality score in the BAM files was calculated and the insert/deletion (InDel) regions were realigned with the Genome Analytic Toolkit (version 3.6). VarDict was used to identify variants against the human genome reference, and the variants were annotated with SnpEff (version 4.2).

### *FPGS R419W* mutation detection by droplet digital PCR (ddPCR)

The ddPCR assays were performed with the Custom TaqMan SNP genotyping assays (ThermoFisher, Waltham, MA) and the 713nt-length PCR fragments with or without *FPGS* R419W were used as the positive or negative control. A seven-point standard curve (0%, 1%, 3%, 5%, 10%, 30%, 100%) was performed as the intra-run control. Background from water instead of DNA added to the reaction mixture was analyzed. This study was performed on a QX200 Droplet Digital PCR system (Bio-Rad), consisting of a T100 Thermalcycler, a QX200 Droplet Generator, and a QX200 Droplet Reader. The PCR reaction mixture (20 μL) contained 10 μL of ddPCR Supermix (no dUTP) for probes, 1 μL of each primer/probe mix (target and reference, labeled with VIC and FAM fluorophores, respectively), and 8 μL of extracted DNA. PCR cycling conditions consisted of 94 °C-30 s, and 60 °C-60 s for 40 cycles and 98 °C for 10 min. Results were analyzed with QuantaSoft v.1.7 software (Bio-Rad) and reported as copies per mL of plasma or % of mutant DNA in the tumor. Details of the primers’ sequences are listed in the supplementary Table 1.

### Site-directed mutagenesis

We generated the *FPGS* mutations *FPGS* R419W, R141H, V136F and K215_V218delinsSP by site-directed mutagenesis on the mammalian expression pFastBac-His vector (ThermoFisher), using the QuikChange Lightning Site-Directed Mutagenesis kit (Agilent) according to the manufacturer’s instructions.

### Enzymatic activity of FPGS

For FPGS enzymatic activity measurement, *FPGS* cDNAs were cloned into a pFastBac expression vector, and mutations were generated with the QuikChange Lightning Site-Directed Mutagenesis kit (Agilent). Wildtype and R419W, R141H, V136F and K215_V218delinsSP viral *FPGS* plasmids were transduced into Sf9 insect cells and the indicated FPGS proteins were purified. FPGS proteins were quantified by comparison with the gradient BSA running on the same PAGE gel. FPGS proteins (2.5/5 ng) were applied to 10 µl reaction buffer :(20 mM Tris (pH8.85), 20 mM MgCl_2_, 20 mM KCl, 10 mM NaHCO_3_, 100 mM 2-mercaptoethanol, 0.5 mg/ml BSA, 5 mM glutamic acid), 5 µl MTXpg1-6(0.4 µM), 2.5 µl ATP(60 µM) and 10 µl phosphate sensor(2x), incubated at 37 °C for 5 ~ 10 min. Fluorescence was read on BioTek Synergy H4 Hybrid Microplate Reader at 450 nM.

The enzymatic activity of FPGS mutant proteins were tested in triplicate and normalized to the FPGS wild type protein, and the results were plotted using Graph Prism (version 7.05). All experiments were conducted independently. The data are expressed as the means ± the standard error (SEM). Group means were compared with Student’s *t* test for two groups. *P* values < 0.05 were considered statistically significant.

## Results

### Identifications of relapse-specific mutations in *FPGS* R419W

Table 1 lists the demographic and clinical characteristics of the eight patients (1.3 to 15.5 years old at relapse) with relapsed B-cell ALL for whom whole-exome sequencing was performed on diagnostic, remission and relapse clinical samples. The complete genetic lists of this eight trio samples were summarized in the supplementary Table 2. The most notable finding in our exome sequence analysis was the identification of relapse-specific mutation in *FPGS*, in a 4-year-old girl with hyperdiploid ALL.

**Table 1:** Characteristics of 8 patients with childhood ALL who received WES.

ID	Age at relapse	Sex	Cytogenetics	Immunophenotypes	Subtype
439	14.8 y/o	M	45,XY,del(6)(q13,q25),der(9),add(9)(p24),del(9)(q13q22),der(9),t(9;9)(p12;q21),-14,t(17;19)(q22;p13)[11/20]/46xy[9/20]	Precursor B-ALL	*TCF3-HLF*
507	9.9y/o	F	46,XX	Precursor B-ALL	*MEF2D-BCL9*
584	7.3y/o	F	52,XX,+3,+10,+14,+17,+21,+X	Precursor B-ALL	Hyperdiploidy
612	15.5y/o	M	48,XY,t(1;19)(q23;p13),dup(6)(921p25),+8,+20[19]/46,XY[1]	Precursor B-ALL	*TCF3-PBX1*
614	5.4y/o	M	47,XY,+10,del(12)(p1?1p1?3)[1]/46,XY[4]	Precursor B-ALL	*ETV6-RUNX1*
658	13.5y/o	F	46,XX,t(1;11)(p32;q23)[20]	Precursor B-ALL	*KMT2A-EPS15*
682	9.6y/o	M	46,XY[5]	Precursor B-ALL	*ETV6-RUNX1*
696	1.3/o	M	46,XY	Precursor B-ALL	Other

**Figure 1:**
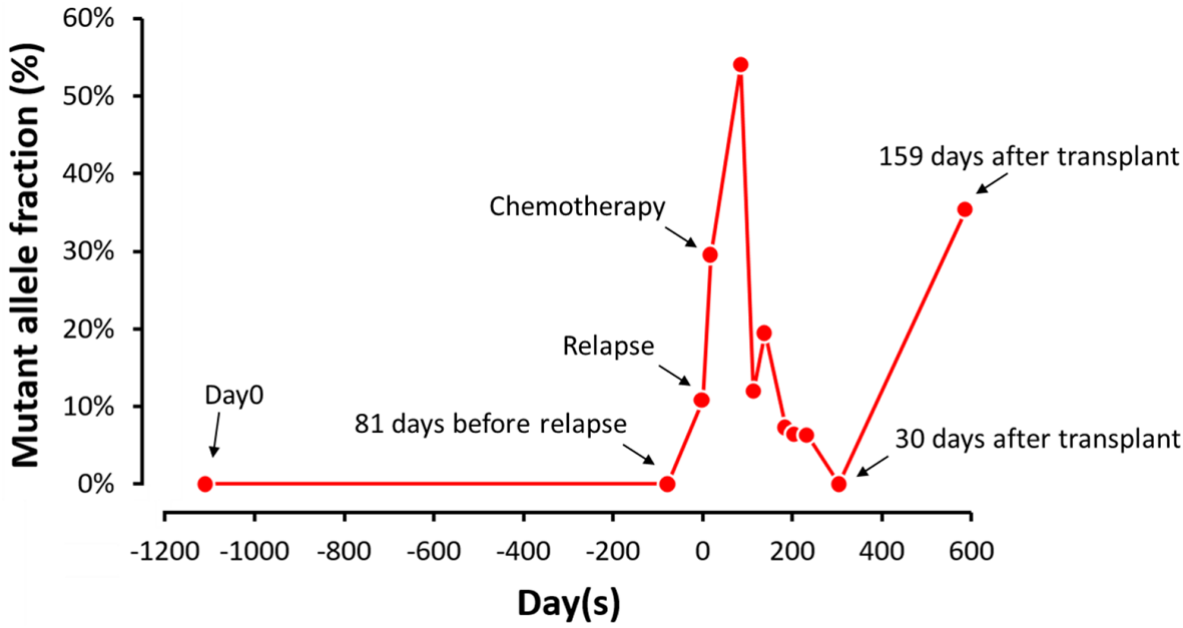
Droplet digital PCR for the follow-up of the patient with *FPGS* R419W. The *FPGS* R419W clone was undetected after stem cell transplantation; however, the clone recurred, and the patient had second relapse.

We design the ddPCR to follow the available clinical samples of the patient with *FPGS* R419W. The *FPGS* mutant was not detected in the diagnostic sample nor in the off-therapy follow-up sample (81 days before her first relapse) with ddPCR. At this point, flow cytometry cannot detect any aberrant clone. ddPCR showed MAF 15% *FPGS* R419W mutant in her first relapse sample and the percentage increased to 55% in agreement with outgrowth of the mutant clone but decreases after the reinduction chemotherapy. The *FPGS* R419W remained undetectable on day 30 after the bone marrow transplantation but became detectable again (MAF 6%) in her second relapse sample on day 159 after transplantation (Fig. 1). The low MAF at first relapse was due to low blast percentage (around 20.5%) at that time.

### Deep sequencing of *FPGS*, *NT5C2* and *PRPS1* in a large series of diagnostic and relapsed clinical samples

To determine the prevalence of the *FPGS* mutations, we studied 372 patients including 299 diagnostic and 73 relapsed samples, of which 58 were paired samples. Table 2 lists the clinical characteristics of these patients. Since MTX and mercaptopurine are two main antileukemic agents used during maintenance therapy in current ALL protocols, we also performed deep sequencing of *NT5C2* and *PRPS1*, two important genes in the thiopurine pathway^16–18^, in this cohort of patients.

**Table 2:** Clinical characteristics of childhood ALL patients in this study.

	Total (N = 380)	Non-Relapsed ALL (N = 299)	Relapsed ALL (N = 81)*
**Age—no. (%)**			
< 1 year	15 (3.9)	9 (3.0)	6 (7.4)
1–9 year	254 (66.8)	207 (69.2)	47 (58.0)
> 10 year	111 (29.2)	83 (27.8)	28 (34.6)
**Sex—no. %**			
Male	220 (58.9)	172 (57.5)	48 (59.3)
Female	160 (42.1)	127 (42.5)	33 (40.7)
**WBC (× 10**^**9**^**/liter)—no. (%)**			
< 50	252 (66.3)	212 (70.9)	40 (49.4)
50–100	41 (10.8)	29 (9.7)	12 (14.8)
> 100	76 (20.0)	52 (17.4)	24 (29.6)
No data	11 (2.9)	6 (2.0)	5 (6.2)
**Subtype—no. (%)**			
T-ALL	59 (15.5)	49 (16.4)	10 (12.3)
11q23	16 (4.2)	9 (3.0)	7 (8.6)
*BCR-ABL1*	13 (3.4)	7 (2.3)	6 (7.4)
*TCF3-PBX1*	17 (4.5)	16 (5.4)	1 (1.2)
*ETV6-RUNX1*	38 (10.3)	32 (10.7)	6 (7.4)
Hyperdiploidy	55 (14.5)	51 (17.1)	4 (4.9)
Hypodiploidy	2 (0.5)	0 (0.0)	2 (2.5)
Other	180 (47.1)	135 (45.2)	45 (55.6)

*This included the eight trio samples submitted to whole exome sequencing.

**Figure 2:**
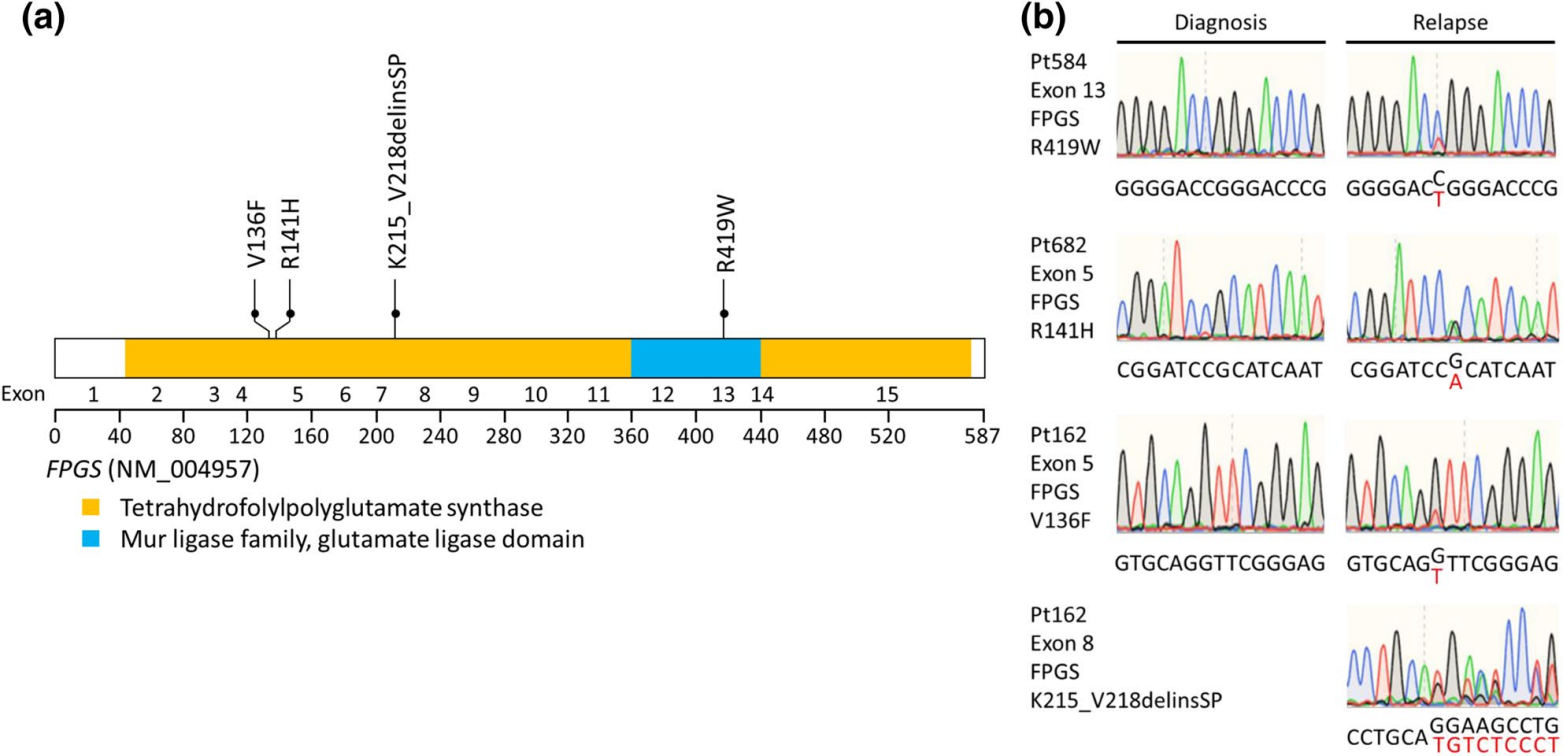
*FPGS* mutations in relapsed pediatric ALL. (**a**) Schematic representation of the structure of the FPGS protein. Tetrahydrofolylpolyglutamate synthase, Mur ligase family, and glutamate ligase domains are indicated. *FPGS* mutations identified in relapsed pediatric samples are shown. Filled circles represent heterozygous mutations. (**b**) DNA sequencing chromatograms of paired diagnosis and relapse genomic ALL DNA samples showing representative examples of relapse-specific heterozygous *FPGS* mutations, with the mutant allele sequence highlighted in red.

We identified two additional patients with three *FPGS* mutants (R141H, V136F and K215_V218delinsSP, the last two mutants occurred in the same patient), as well as six patients with *NT5C2* and two patients with *PRPS1* mutants. One patient had *NT5C2* and *PRPS1* mutants concurrently with different MAF. All these mutants were validated by Sanger sequencing except case ID17 with *NT5C2* and *PRPS1* mutants because of low MAF 8% for *NT5C2* and 6% for *PRPS1* (supplementary Table 3). All *FPGS* mutations were listed in Fig. 2 and were not previously reported. The *NT5C2* and *PRPS1* mutations were listed in the supplementary Figs. 1 and 2, respectively. The details of MAF and clinical outcomes for *FPGS*, *NT5C2* and *PRPS1* mutations were listed in the supplementary Table 3. The patient with *BCR-ABL1* fusion and *PRPS1* mutations and the other with T-ALL and *NT5C2* mutation both had very short initial remission duration of 6 months (supplementary Table 3). The other patient who had both *PRPS1* and *NT5C2* mutations also had short remission of 15 months. The remaining 6 patients with either *NT5C2* or *FPGS* mutations had relatively long duration of initial remission (21 to 37 months).

### Relapse-specific *FPGS* mutations impair MTX metabolism and lead to drug resistance

**Figure 3:**
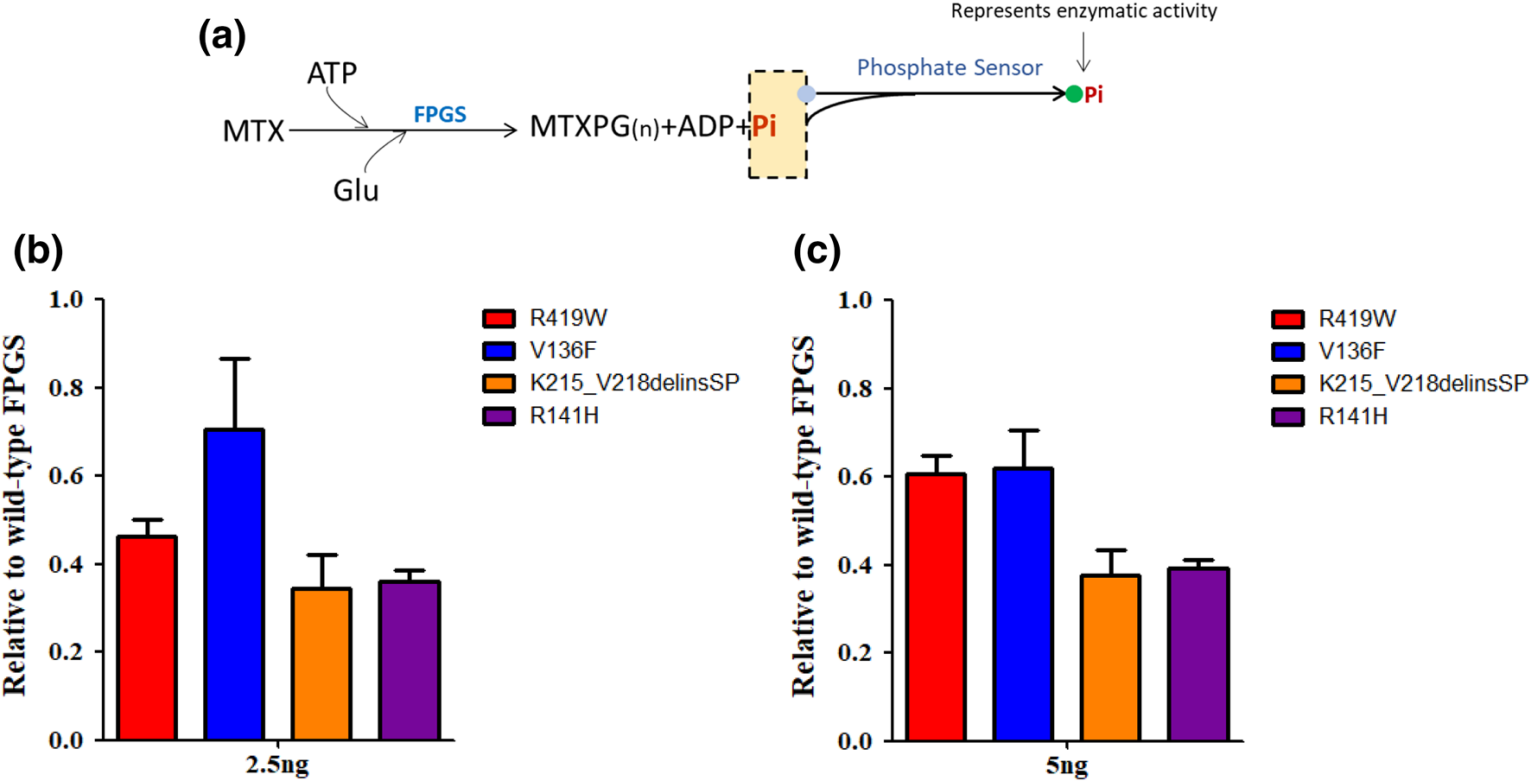
Effects of *FPGS* mutation on polyglutamation enzymatic activity using methotrexate as the substrate. (**a**) FPGS activity was measured by quantifying ATP consumption with a phosphate sensor kit. Wild-type or mutant FPGS proteins were expressed and purified to homogeneity. Mutant FPGS proteins exhibited 30% to 66% reduction in activity under the same experimental conditions. (**b**) FPGS proteins 2.5 ng (**c**) FPGS proteins 5.0 ng.

Given the established role of FPGS in MTX metabolism, we hypothesized that *FPGS* mutations would be directly associated with MTX resistance. We therefore produced all four mutant FPGS proteins using the SF9 cell system and directly compared their enzymatic activity with wild-type FPGS. When incubated with the wild-type protein, MTX was activated efficiently through polyglutamation in vitro across a range of FPGS concentrations, as measured by the release of phosphate signal. In contrast, mutant FPGS proteins exhibited around 30–66% reduction in activity under the same experimental conditions. Because MTX polyglutamates is the main metabolite responsible for the antileukemic effects of this drug, we concluded that reduced polyglutamylation due to loss-of-function FPGS mutation directly confers to resistance to MTX (Fig. 3).

## Discussion

Studies focusing on the relapsed childhood ALL samples have identified several pathways that are highly susceptible to mutation, including those involved in RAS signaling, JAK-STAT signaling, transcriptional factors of lymphoid development, nucleoside metabolism, epigenetics modification and cell cycle regulation^13,14,30^. In this study, we identified four acquired relapse-specific mutations in *FPGS* in three children with relapsed ALL. Functional studies showed that these mutations resulted in impairment of MTXPGs production, one of the main drugs in the treatment of childhood ALL^2,31^. Thus, *FPGS* relapse-specific mutations represent a pharmacogenetic pathway related to childhood ALL in relapse.

Once taken up by cells, MTX undergoes polyglutamylation catalyzed by FPGS, which sequentially adds up to 10 glutamate residues to the γ-carboxyl residue of MTX^32^. This unique metabolism retains MTX intracellularly and increases its cytotoxic activity. The ability of leukemic cells to accumulate MTX polyglutamates ex vivo has been shown to be a determinant of MTX activity in childhood ALL patients^33^. Indeed, higher accumulation of long-chain polyglutamates of MTX has been associated with better overall and event-free survival in patients with ALL^34,35^. The loss of FPGS activity is an established mechanism of resistance to MTX polyglutamylation both in vitro and in vivo^33,36–38^, while RNA splicing leading to different isoform of *FPGS* was a mechanism leading to MTX resistance in ALL^37^. However, the relapse-specific *FPGS* mutations were not identified among several diagnosis-relapse pair studies focusing upon the relapse-driven mutations^13,16,17,39^. Despite many comprehensive genomic studies have focused on the diagnostic somatic mutations, no *FPGS* mutations are found in newly diagnosed patients. A recent study by Schroeder et al.^40^ used a comprehensive multi-omics dataset including DNA-sequencing, RNA-sequencing, DNA methylation array and proteome MASS-spec data from matched diagnosis and relapse samples adult and pediatric patients with B-cell ALL. They identified acquired *FPGS* alterations in six patients, of whom four had *FPGS* deletions, one *FPGS* deletion and point mutation, and one *FPGS* point mutation only^40^. These three mutations (P347S, P367L, G417E) were in or close-by the annotated Mur-ligase domain related to ATP-binding and ligase activity, which is close to the R417W we identified. During the preparation of this manuscript, another study from China also identified eight *FPGS* mutants (8/103, 7.8%) in a relapsed childhood ALL cohort^30^. Four mutations (R369C, G370V, G417R and P421L) were clustered in the glutamate ligase domain. Four other mutations (E115K, K167T, D195H, and R558 > PGES) occurred outside the active site. These *FPGS* mutants were also functionally validated. This cohort is also of Chinese origin. These studies showed that *FPGS* mutations were recurrent genetic events in relapsed childhood ALL.

Relapse-specific genes mutations in the mercaptopurine pathway, such as *NT5C2* and *PRPS1*, have been demonstrated in several studies^13,16–18,30^. We identified two patients with *PRPS1* mutations, three patients with *NT5C2* mutants and one with mutations of both genes in the relapsed samples. *PRPS1* mutations were first identified in relapsed ALL samples in a Chinese cohort but rarely identified in a German cohort^18^. Ding et al^39^ performed exome sequencing and targeted sequencing for 240 childhood ALL cases in Asian patients, and identified a single *NT5C2* mutation in their relapse samples, but no *PRPS1* or *FPGS* mutations. The basis of the differences in frequency of *PRPS1* and *NT5C2* mutations in these studies remains unknown^41^. Conceivably, the low frequency of the two early studies was due to small numbers of relapsed patients studied (16 and 40 patients, respectively)^18,39^. The higher frequency in our study (8.2%) may be related to long duration of low-dose continuation treatment in our patient cohort (146 weeks for boys and 120 weeks for girls), allowing chemotherapy-induced mutations to develop, a finding recently reported by Li et al.^33^. In fact, the result of our study is consistent with that of the recent study from China in which the prevalence rate of *PFGS*, *NT5C2*, *PRPS1* was 8.7, 7.6 and 5.5% in relapsed childhood ALL, respectively^30^. Indeed, novel strategy targeting the dysregulated purine pathway of *NT5C2* mutations might prove worthwhile in future clinical trials^42^. To this end, strategy targeting the *FPGS* mutations which cause inadequate polyglutamylation of MTX deserves further investigations.

In conclusion, besides mutations affecting the mercaptopurine pathway, *FPGS* relapse-specific mutations should be considered a cause of relapse in childhood ALL.

## Supplementary information

Supplementary information

Supplementary table 2
