# Supplementary material for: FPGS relapse-specific mutations in relapsed childhood acute lymphoblastic leukemia
Source: Sci Rep. 2020 Jul 21;10:12074. doi: 10.1038/s41598-020-69059-y (PMC7374087; doi:10.1038/s41598-020-69059-y)
Supplement: Supplementary file 1 — Supplementary information [file 41598_2020_69059_MOESM1_ESM.pdf]

FPGS relapse-specific mutations in relapsed childhood acute lymphoblastic leukemia

Sung-Liang Yu[1,2,3,4,5,6*], Hui Zhang[7*], Bing-Ching Ho[1], Chih-Hsiang Yu[2], Chia-Ching Chang[2], Yin-Chen Hsu[2], Yu-Ling Ni[3], Kai-Hsin Lin[8], Shiann-Tarng Jou[8], Meng-Yao Lu[8], Shu-Huey Chen[9], Kang-Hsi Wu[10], Shih-Chung Wang[11], Hsiu-Hao Chang[8], Ching-Hon Pui[12], Jun J Yang[13], Jinghui Zhang[14], Dong-Tsamn Lin[3,8], Shu-Wha Lin[2], Xiaotu Ma[14], Yung-Li Yang[3,8]

[1]Centers of Genomic and Precision Medicine, National Taiwan University, Taipei, Taiwan

[2]Department of Clinical Laboratory Sciences and Medical Biotechnology, College of Medicine, National Taiwan University, Taipei, Taiwan

[3]Department of Laboratory Medicine, National Taiwan University Hospital and National Taiwan University College of Medicine, Taipei, Taiwan;

[4]Institute of Medical Device and Imaging, College of Medicine, National Taiwan University, Taipei, Taiwan

[5]Graduate Institute of Pathology, College of Medicine, National Taiwan University, Taipei, Taiwan

[6]Graduate Institute of Clinical Medicine, National Taiwan University College of Medicine, Taipei, Taiwan

[7]Department of Hematology & Oncology, Guangzhou Women and Children's Medical Center, Guangzhou, Guangdong, China

[8]Department of Pediatrics, National Taiwan University Hospital and National Taiwan University College of Medicine, Taipei, Taiwan;

[9]Department of Pediatrics, Taipei Medical University–Shuang Ho Hospital, Taipei, Taiwan;

[10]Division of Pediatric Hematology &Oncology, China Medical University Children’s Hospital, Taichung, Taiwan;

[11]Department of Pediatrics, Changhua Christian Hospital, Changhua, Taiwan.

[12]Department of Oncology, St. Jude Children's Research Hospital, Memphis, Tennessee, USA

[13]Department of Pharmaceutical Sciences, St. Jude Children’s Research Hospital, Memphis, Tennessee, USA

[14]Department of Computational Biology, St. Jude Children's Research Hospital, Memphis, Tennessee, USA

*These authors contributed equally to this work: Sung-Liang Yu and Hui Zhang

Corresponding author:

Yung-Li Yang

Department of Laboratory Medicine, National Taiwan University Hospital and National Taiwan University College of Medicine, Taipei, Taiwan;

No. 7, Chung Shan S. Rd, Zhongshan S. Rd, Zhongzheng Dist, Taipei 100, Taiwan, R.O.C.

Tel.: +886-2-23123456-71712

Fax:+886-2-23224263

E-mail: yangyl92@ntu.edu.tw

Supplementary Table 1. Primer sequences

	Sequence (5’ to 3’)
**Custom TaqMan SNP assay**	
FPGS_R419W forward primer	TTCGAGTCTTGCTCTTCAATGCTA
FPGS_R419W reverse primer	TCACCTGCAGCAGCTTCA
Reporter 1-FAM-NFQ	CCGGGTCCCGGTCC
Reporter 1- VIC-NFQ	CCGGGTCCCAGTCC
**Artificial amplicon**	
FPGS-F	GCCCCTCACCTGGTACCTG
FPGS-R	AGAGGCAAACTGAGGCTCGG
FPGS-mF	GCCGCCGGGTCCCaGTCc
FPGS-mR	GGACtGGGACCCGGCGGC
**Sanger validation**	
FPGS_R419W F	GGTCCTGAGTGTTGAGGGCGG
FPGS_R419W R	AGGGGGAGGGGATTGGCACC
FPGS_R141H/V136F F	GGGGCACCAGGAACAAACCG
FPGS_R141H/V136F R	ACCTCCACCACTGCCAGGTC
FPGS_K215_V218delinsSP F	GCGGGGCTTATGACTGCACC
FPGS_K215_V218delinsSP R	TCCAGTCACACAGTGAAGCCAGG
NT5C2_R39Q F	TCAAACAGCATGTCGTGTTATACATC
NT5C2_R39Q R	GCTTCTGGCAGCCAAATACA
NT5C2_R238W F	AGGTTCCCCCATTCCTGTTGTGG
NT5C2_R238W R	AAGGGGTGTGACTGCTCAAGTTT
NT5C2_R367Q F	GCTCTGGTCAGCACAGTGGAGC
NT5C2_R367Q R	CCTGCCTTTTGACCACCTCTGACT
PRPS1_P106A F	TCTGGGTACCATAGTGCCTTTAACA
PRPS1_P106A R	ACTGCCTCCCTATCTAACCACCTG
PRPS1_A190T F	TATTCTCCTCCCCAAAACAAGCCCA
PRPS1_A190T R	TACCAGCCCCATCAATCCACACTTA

Supplementary Table 3. Time to relapse, overall survival and MAF of relapsed childhood ALL patients with *FPGS*, *NT5C2* or *PRPS1* mutations

**ID**	**Sex**	**Age at D0**	**Cytogenetics**	**Subtype**	**Mutation (MAF)**	**Time to relapse (month)**	**Overall survival (month)**	**Outcome**
584	F	4.16	52,XX,+3,+10,+14,+17,+21,+X	Hyperdiploidy	*FPGS*_p.R419W(15%)	37	57	Deceased
662	M	7.02	46,XY[5]	*ETV6-RUNX1*	*FPGS*_p.R141H(37%)	21	33	Deceased
162	M	4.16	48,XY, +der(7)t(7;22)(p13;q11), dic(9:20)(p11;q11),+21,-22, +mar1,+mar2[5]/49,idem,+21[1]/46,XY[2]	Other	*FPGS*_p.V136F(29%); *FPGS*_p.K215_V218delinsSP(43%)	31	66	Deceased
196	F	3.50	i8q,-15q,-12p,i17q	Other	*NT5C2*_p.R39Q(11%)	24	35	Deceased
434	F	9.50	46,XX[18]/47,XX,+8[2]	*TCF3-ZNF384*	*NT5C2*_p.R39Q(26%)	33	38	Deceased
544	F	7.50	46,XX[8]	Other	*NT5C2*_p.R39Q(20%)	29	41	Deceased
717	M	13.14	No mitosis	T-ALL	*NT5C2*_p.R367Q(41%)	6	12	Deceased
17	M	2.28	46,XY[20]	Other	*NT5C2*_p.R238W(8%); *PRPS1*_p.P106A(6%)	15	23	Deceased
68	M	7.56	46,XY,t(9;22)(q34;q11)[6]/46,XY[2]/ 45,XY,idem, der(20)t(20;21)(q13;q11),-21[2]	*BCR-ABL1*	*PRPS1*_p.A190T(13%)	6	20	Deceased

(a)

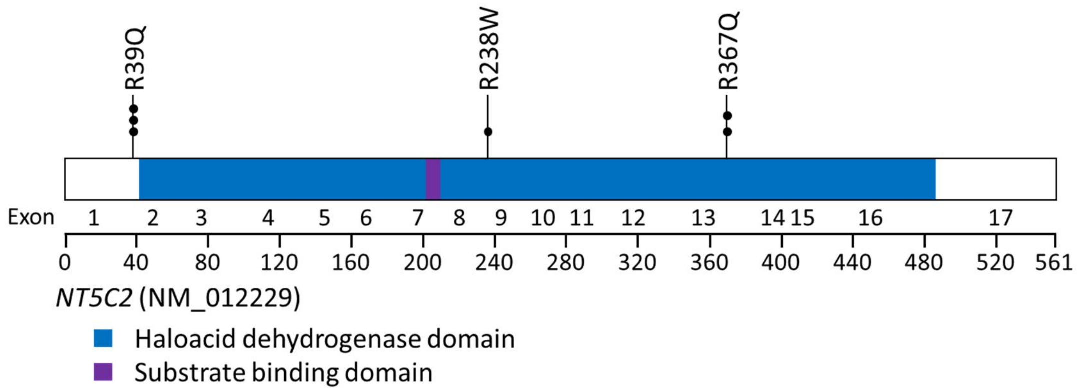

(b)

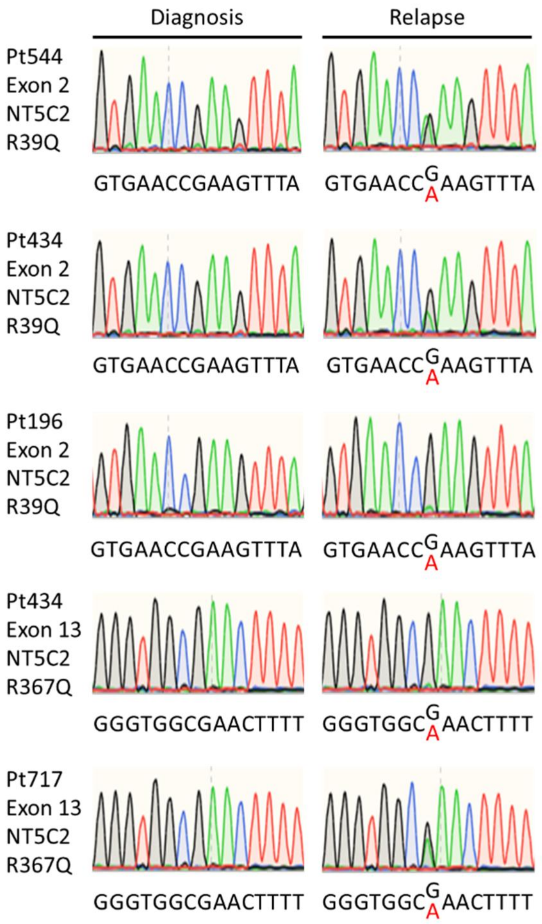

Supplementary Figure 1. *NT5C2* mutations in relapsed pediatric ALL. (**a**) Schematic representation of the structure of the NT5C2 protein. The haloacid dehydrogenase domain and substrate binding domain are indicated. *NT5C2* mutations identified in relapsed pediatric samples are shown. Filled circles represent heterozygous mutations. (**b**) DNA sequencing chromatograms of paired diagnosis and relapse genomic ALL DNA samples showing representative examples of relapse-specific heterozygous *NT5C2* mutations, with the mutant allele sequence highlighted in red.

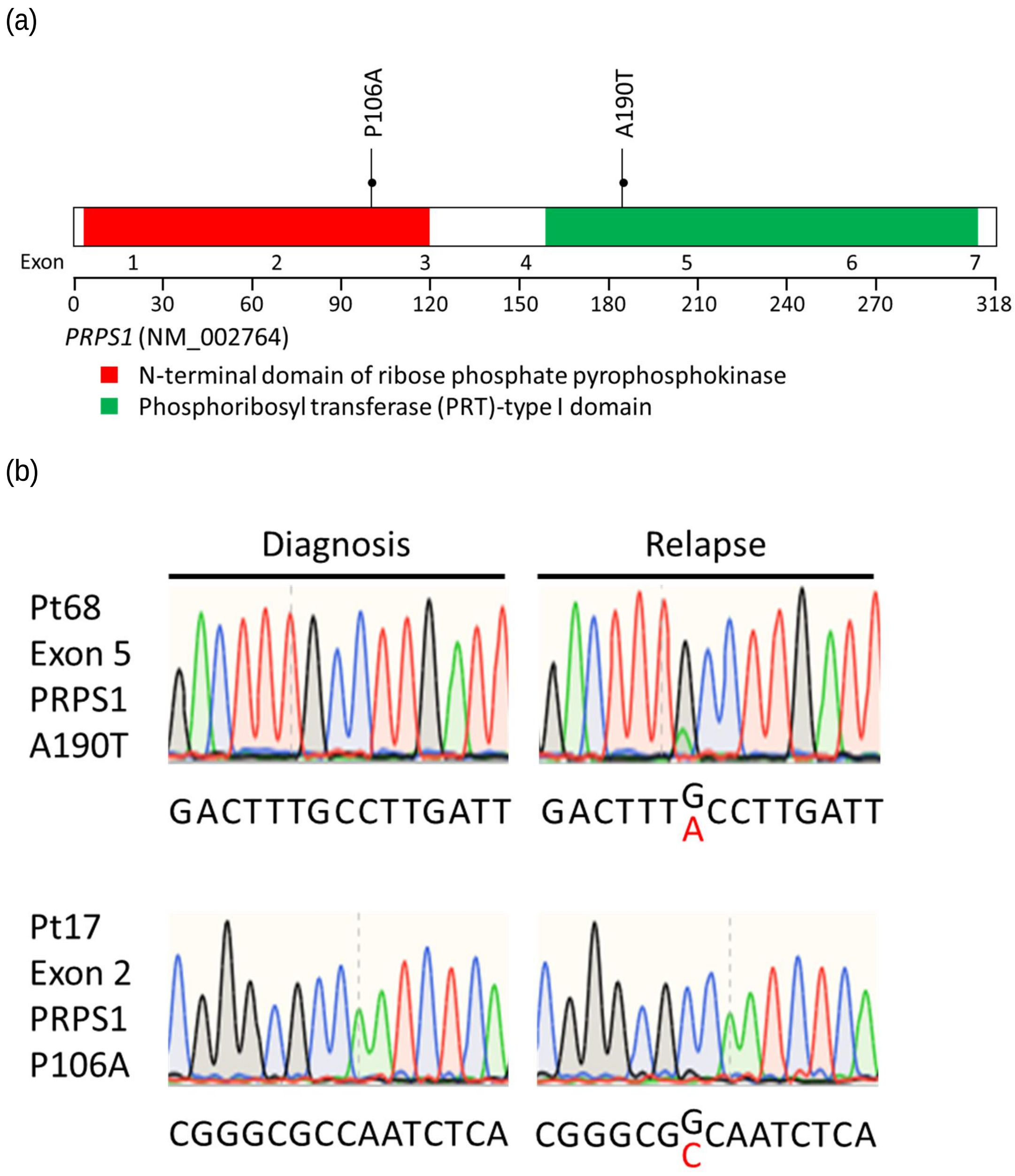

Supplementary Figure 2. *PRPS1* mutations in relapsed pediatric ALL. (**a**) Schematic representation of the structure of the PRPS1 protein. The N-terminal domain of ribose phosphate pyrophosphokinase and phosphoribosyl transferase (PRT)-type I domain are indicated. *PRPS1* mutations identified in relapsed samples are shown. Filled circles represent heterozygous mutations. (**b**) DNA sequencing chromatograms of paired diagnosis and relapse genomic ALL DNA samples showing representative examples of relapse-specific heterozygous *PRPS1* mutations, with the mutant allele sequence highlighted in red.
